# Mutations in the Autoregulatory Domain of β-Tubulin 4a Cause Hereditary Dystonia

**DOI:** 10.1002/ana.23832

**Published:** 2013-02-19

**Authors:** Joshua Hersheson, Niccolo E Mencacci, Mary Davis, Nicola MacDonald, Daniah Trabzuni, Mina Ryten, Alan Pittman, Reema Paudel, Eleanna Kara, Katherine Fawcett, Vincent Plagnol, Kailash P Bhatia, Alan J Medlar, Horia C Stanescu, John Hardy, Robert Kleta, Nicholas W Wood, Henry Houlden

**Affiliations:** 1 Department of Molecular Neuroscience, University College London Institute of Neurology and National Hospital for Neurology and NeurosurgeryLondon, United Kingdom; 2 Department of Neurology and Laboratory of Neuroscience, IRCCS Istituto Auxologico ItalianoUniversita degli Studi di Milano, Italy; 3 Department of Womens Health, UCLHLondon, United Kingdom; 4 Centre for Nephrology, University College LondonLondon, United Kingdom; 5 Department of Genetics, King Faisal Specialist Hospital and Research Centre, PO Box 3354Riyadh 11211, Saudi Arabia

## Abstract

Dystonia type 4 (DYT4) was first described in a large family from Heacham in Norfolk with an autosomal dominantly inherited whispering dysphonia, generalized dystonia, and a characteristic hobby horse ataxic gait. We carried out a genetic linkage analysis in the extended DYT4 family that spanned 7 generations from England and Australia, revealing a single LOD score peak of 6.33 on chromosome 19p13.12-13. Exome sequencing in 2 cousins identified a single cosegregating mutation (p.R2G) in the β-tubulin 4a (TUBB4a) gene that was absent in a large number of controls. The mutation is highly conserved in the β-tubulin autoregulatory MREI (methionine–arginine–glutamic acid–isoleucine) domain, highly expressed in the central nervous system, and extensive in vitro work has previously demonstrated that substitutions at residue 2, specifically R2G, disrupt the autoregulatory capability of the wild-type β-tubulin peptide, affirming the role of the cytoskeleton in dystonia pathogenesis.

Primary torsion dystonias (PTDs) are a group of disorders characterized by involuntary muscle contractions affecting 1 or more sites of the body, resulting in twisting and repetitive movements or abnormal postures.^1^,^2^ There is a wide phenotypic spectrum associated with PTDs, which often show significant intrafamilial variability.^3^–^5^ PTDs can be generalized or focal and have either an early or a late onset.^6^ To date, 6 autosomal dominant (dystonia type [DYT] 1, 4, 6, 7, 13, and 21) loci and 2 autosomal recessive (DYT2 and 17) loci have been identified.^7^–^16^ Three genes with autosomal dominant inheritance have been determined so far. DYT1 is caused by mutations in *TOR1A*,^13^ and DYT6 is caused by mutations in *THAP1*^8^ and *CIZ1*^17^ on chromosome 9q34.

DYT4 was first described in 1985 by forensic psychiatrist Neville Parker^15^ in a large family with third decade onset of autosomal dominantly inherited whispering dysphonia and generalized dystonia. More than 30 affected individuals have been reported, typically presenting with a laryngeal dysphonia progressing to a generalized dystonia with a peculiar “hobby horse” ataxic gait. The family originally descended from an affected male who was born in 1801 in the small rural coastal town of Heacham in Norfolk. He had 9 children. Several lived in the Heacham and Dersingham area; 1 likely affected son had 3 affected daughters, and 2 of them emigrated in 1886 to Townsville, Australia. Several affected and unaffected family members remain in England and Australia, some distantly related; however, no other similar kindred have so far been described worldwide with this phenotype. Linkage analysis in this family has excluded known dystonia loci.^15^,^18^–^22^ A recently published review of the surviving Australian affected family members provided additional information about the family and disease phenotype, indicating frequent progression to generalized and gait dystonia, with 5 of 9 cases exhibiting the unusual gait pattern described by Parker.^21^

We report the identification of genetic linkage in this family to chromosome 19p13.12-13 with a highly significant LOD score of 6.33. We subsequently carried out exome sequencing in 2 affected cousins to identify a single mutation in the autoregulatory MREI (methionine–arginine–glutamic acid–isoleucine) domain of the β-tubulin-4a (*TUBB4a*) gene. This conserved mutation is highly likely to be pathogenic based on the segregation, absence in a large number of controls, expression data, and the proven effect of β-tubulin MREI domain mutations.

## Subjects and Methods

**FIGURE 1:**
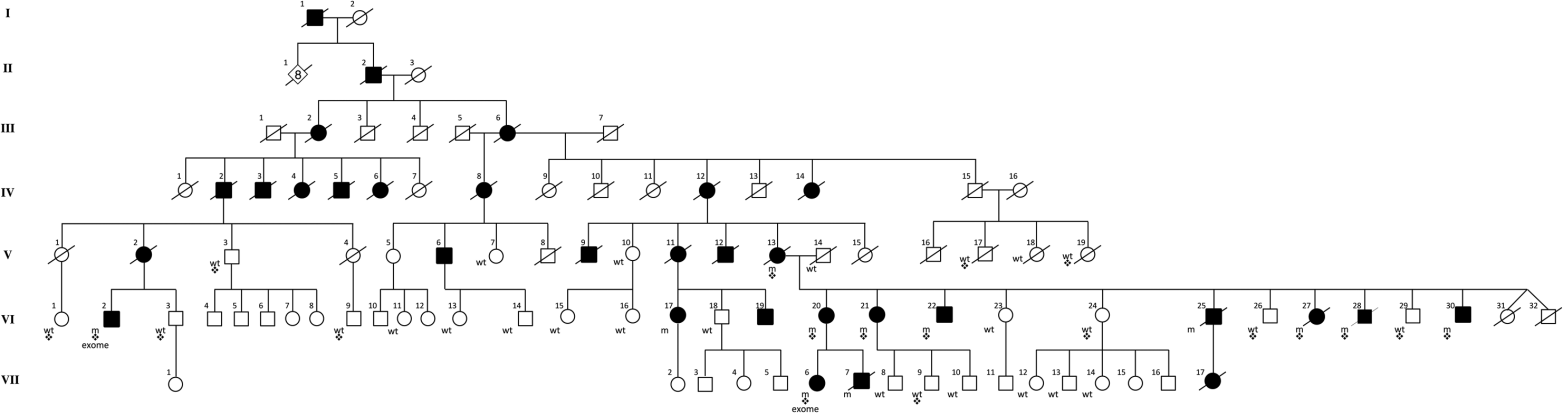
Pedigree of the dystonia type 4 family. Where mutation screening has been performed, individuals are marked with either wt (wild-type allele) or m (R2G heterozygote); exome indicates exome sequencing performed. The symbols (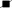, 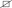, 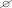) indicates individuals included in linkage analysis. VI-27 and VI-28 were known to have Wilson disease and were also heterozygous for the R2G variant.

Blood samples were collected and DNA extracted with informed consent from 38 family members from the original extended family seen by Neville Parker (marked with either wt or m on the pedigree in Fig 1). Clinical details of affected family members have been reported previously.^15^,^20^,^21^ Linkage to known loci was previously excluded, and sequencing of the *TOR1A* and *THAP1* genes was negative. Table 1 details the clinical features of selected affected family members, obtained through direct patient interview and review of historical patient records. Exome sequencing was performed on 2 cases (VI-2 and VII-6). An additional 95 UK dystonia families and 75 dystonia brains cases were further analyzed and found negative. This mutation was absent from 1,045 UK control individuals and absent in 7,203 exomes from the University College London (UCL) and National Heart, Lung, and Blood Institute exome sequencing projects.

**TABLE 1:** Clinical Characteristics of Selected Affected Family Members

Patient ID	Age at Onset, yr	Age at Examination, yr	Clinical Characteristics
V-16	30	44	Dysphonia, progressing over 2 years until patient unable to speak; cervical dystonia (35 years)

V-2	21	42	Dysphonia (rapid progression resulting in psychiatric referral); swallowing difficulties (25 years), cervical dystonia (34 years), gait affected (37 years)

V-24	23	31	Dysphonia; progression over 6 years to involve cervical muscles, tongue, followed by limb dystonia

V-14	37	60	Onset with stooped posture; progressive dysphonia with swallowing difficulties over 5 years; cervical and oral dystonia; wheelchair bound (53 years)

V-26	13	29	Severe dystonic gait, hepatitis, and hemolytic anemia; KF rings; ataxia; diagnosed as having Wilson disease but with additional dystonic features typical of DYT4

V-27	15	29	Dysarthria; KF rings; upper limb dystonia; diagnosed as having Wilson disease but with additional dystonic features typical of DYT4

V-20	28	37	Cervical dystonia; progressive dysphonia (30 years); no swallowing difficulties; left hemidystonia (32 years)

V-18	13	35	Dysphonia; cervical dystonia (14 years)

The patient ID refers to the position of the individual on the family tree as per Figure 1.

DYT4 = dystonia type 4; KF = Kayser–Fleischer.

Exome sequencing and interpretation methods, family mutation screening, and expression analysis details^23^ are given in detail in the Supplementary Methods.

### Genetic Linkage Analysis

This was carried out on 19 family members (marked on pedigree in Fig 1), comprising 10 unaffected and 9 affected cases. These were genotyped using Illumina CytoSNP12 arrays with 301,232 genome-wide single nucleotide polymorphism (SNP) markers, and the raw data processed using GenomeStudio software (Illumina, San Diego, CA). Genotypes were examined with the use of multipoint parametric linkage analysis, and haplotype reconstruction was performed with Simwalk2.^24^ There were 24,000 informative SNPs, equally spaced 0.1cM apart, used in the analysis. Genotype data were formatted for Simwalk2 using Mega2 (version 4.0)^25^ via ALOHOMORA.^26^ Mendelian inconsistencies were checked with PedCheck (version 1.1).^27^ An autosomal dominant model was specified with an estimated allele frequency of 0.0001 and 90% penetrance. The linkage region identified was subsequently used to filter the genetic variants obtained from exome sequencing.

## Results

**FIGURE 2:**
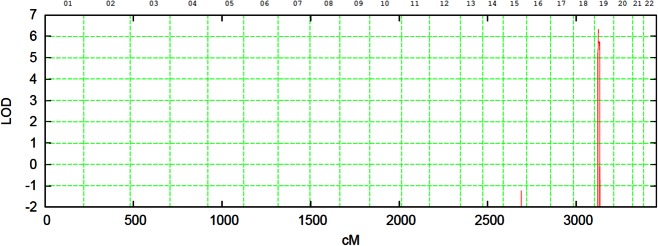
Multipoint parametric linkage analysis of the kindred indicating a single linkage peak at 19p13.3 with an LOD score of 6.33. [Color figure can be viewed in the online issue, which is available at http://www.annalsofneurology.org.]

Linkage to the known DYT genetic loci was excluded by multipoint parametric linkage analysis, which identified, across the whole autosomal genome, a single significant linked region on chromosome 19p13.12-13 between SNP markers rs12977803 and rs2303099. The maximum LOD score between these markers was 6.33. There is some historical evidence in the first and second generation of the family tree of male-to-male transmission, and there were no other regions of linkage in the genome (Fig 2).

Exome sequencing was performed on 2 affected cousins. A summary of the data can be found in Table 2. Following alignment and quality assessment of the data, 23398 and 23701 variants were identified in exome data for VI-2 and VII-6, respectively. The filtering strategy undertaken initially excluded homozygous and synonymous variants. Variants were then filtered against several control data sets, including the 1000 Genomes, Exome Variant Server, UCL Exome, and cg69 databases, but not dbSNP because of concerns about pathological SNPs being uploaded to it.^28^ In total, 153 and 156 novel variants were identified in the 2 respective patients, 3 of which were located within the linkage region. Both individuals shared 2 novel variants within the linkage region (see Table 2, Fig 1): *TUBB4a* (c.4C>G, p.R2G) and *FCER2* (c.947C>T, p.S316F).

**TABLE 2:** Results from Exome Sequencing of Patients V-2 and VI-6 with the Variants That Were Identified

Variants	Patient	
	V-2	VI-6
Unique reads	136,291,642	137,233,190

Aligned reads, %	86.2	85.4

Mean depth	102	106

Total variants	23,398	23,701

Heterozygous variants	14,333	14,207

Excluding synonymous variants	7,294	7,119

Novel variants	153	156

Variants in linkage region	3	3

Shared variants	2	2

The patient ID refers to the position of the individual on the family tree as per Figure 1.

**FIGURE 3:**
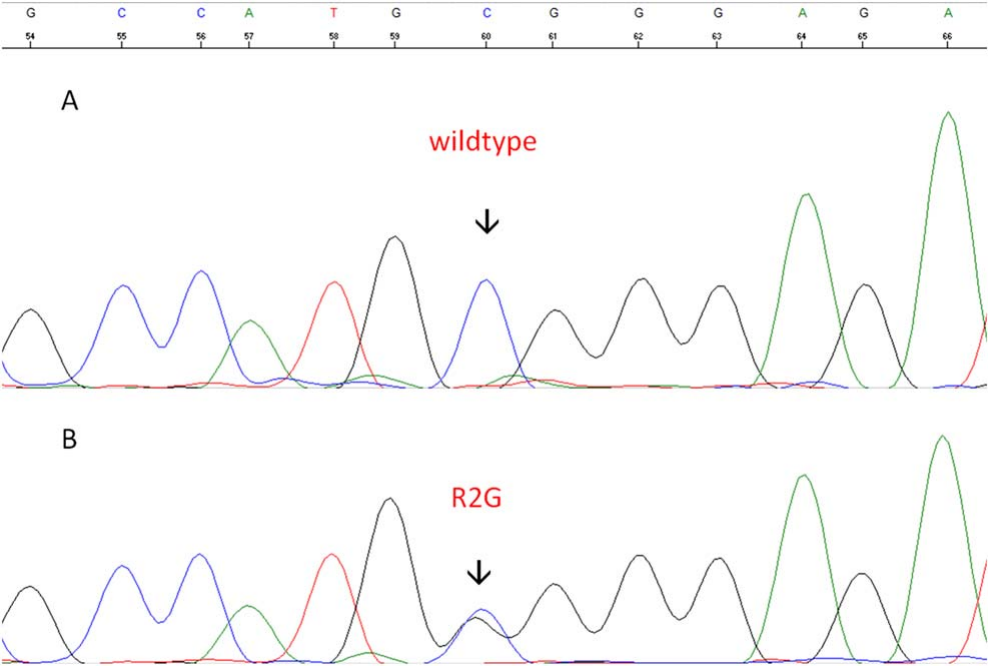
Sequence chromatogram showing (A) an unaffected family member with the wild-type sequence and (B) an affected family member with a heterozygous c.4C>G: p.R2G mutation. [Color figure can be viewed in the online issue, which is available at http://www.annalsofneurology.org.]

The *TUBB4a* variant is located in the highly conserved autoregulatory MREI domain in exon 1 of *TUBB4a* and results in an arginine to glycine (p.R2G) amino acid substitution (Fig 3). The R2G mutation cosegregated perfectly with the disease phenotype, with all affected individuals having the R2G variant. The genotypes of individuals screened in the segregation analysis are indicated in Figure 1 with either wt (wild type) or m (mutant allele: c.4C>G, p.R2G heterozygote). There were no known unaffected carriers, although individuals younger than 18 years were not analyzed. This variant was not found in any of the reference SNP, exome, or in-house exome databases queried, and was not present in 1,045 ethnically matched UK control individuals. The variant was predicted by in silico analysis (SIFT^29^ and PolyPhen2^30^) to be deleterious and was highly conserved in multispecies (Table 3) and multitubulin alignment (Table 4). *FCER2,* however, codes for a low-affinity immunoglobulin E receptor involved in allergy and resistance to parasites. The *FCER2* variant did not segregate with the disease.

**TABLE 3:** Multispecies Protein Sequence Alignment of β-Tubulin Showing the Highly Conserved MREI Subsequence

Species	Protein Sequence Alignment, β-Tubulin
*Homo* (human)	**MREI**VHLQAGQCGNQIGAKFWEVISDEHGIDPTGTYHGD

*Macaca* (macaque)	**MREI**VHLQAGQCGNQIGAKFWEVISDEHGIDPTGTYHGD

*Bos* (cow)	**MREI**VHIQAGQCGNQIGAKFWEVISDEHGIDPTGTYHGD

*Mus* (mouse)	**MREI**VHIQAGQCGNQIGAKFWEVISDEHGIDPTGTYHGD

*Xenopus* (frog)	**MREI**VHLQAGQCGNQIGAKFWEVISDEHGIDPTGAYHGD

*Arabidopsis* (cress)	**MREI**LHIQGGQCGNQIGSKFWEVICDEHGIDSTGRYSGD

Dictyosteliida (slime mold)	**MREI**VQIQAGQCGNQIGSKFWEVISEEHGIQSDGFHAGG

**TABLE 4:** Conservation of the Protein Sequences in the Different Tubulin Isotypes

Tubulin Isotype	Tissue Specificity	Protein Sequence Alignment (human)
*TUBB4a*	Brain-specific	**MREI**VHLQAGQCGNQIGAKFWEVISDEHGIDPTGTYH

*TUBB*	Ubiquitous	**MREI**VHIQAGQCGNQIGAKFWEVISDEHGIDPTGTYH

*TUBB1*	Hematopoietic cells	**MREI**VHIQIGQCGNQIGAKFWEMIGEEHGIDLAGSDR

*TUBB2a*	Brain-specific	**MREI**VHIQAGQCGNQIGAKFWEVISDEHGIDPTGSYH

*TUBB2b*	Brain-specific	**MREI**VHIQAGQCGNQIGAKFWEVISDEHGIDPTGSYH

*TUBB3*	Neuron-specific	**MREI**VHIQAGQCGNQIGAKFWEVISDEHGIDPSGNYV

*TUBB4b*	Ubiquitous	**MREI**VHLQAGQCGNQIGAKFWEVISDEHGIDPTGTYH

*TUBB6*	Ubiquitous	**MREI**VHIQAGQCGNQIGTKFWEVISDEHGIDPAGGYV

*TUBB8*	Ubiquitous	**MREI**VLTQIGQCGNQIGAKFWEVISDEHAIDSAGTYH

The **MREI** subsequence can be seen at the beginning of each sequence.

**FIGURE 4:**
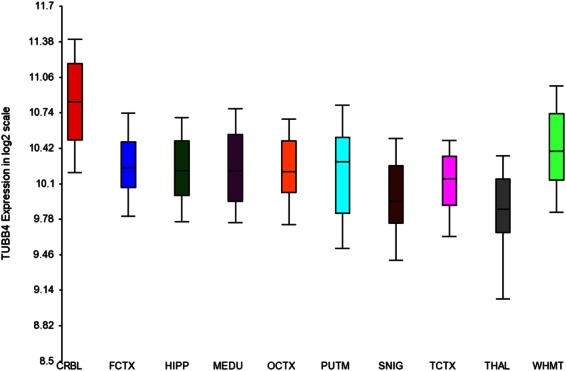
Graph of the expression of the *TUBB4a* gene in 10 brain regions from 134 normal individuals, assessed using the Affymetrix Exon 1.0 ST Array. The level of TUBB4a is given as a log scale with range bars. This showed very high expression in the cerebellum and in the brain overall. The following areas were studied: cerebellum (CRBL), frontal cortex (FCTX), hippocampus (HIPP), medulla (MEDU), occipital cortex (OCTX), putamen (PUTM), substantia nigra (SNIG), temporal cortex (TCTX), thalamus (THAL), and white matter (WHMT). [Color figure can be viewed in the online issue, which is available at http://www.annalsofneurology.org.]

**FIGURE 5:**
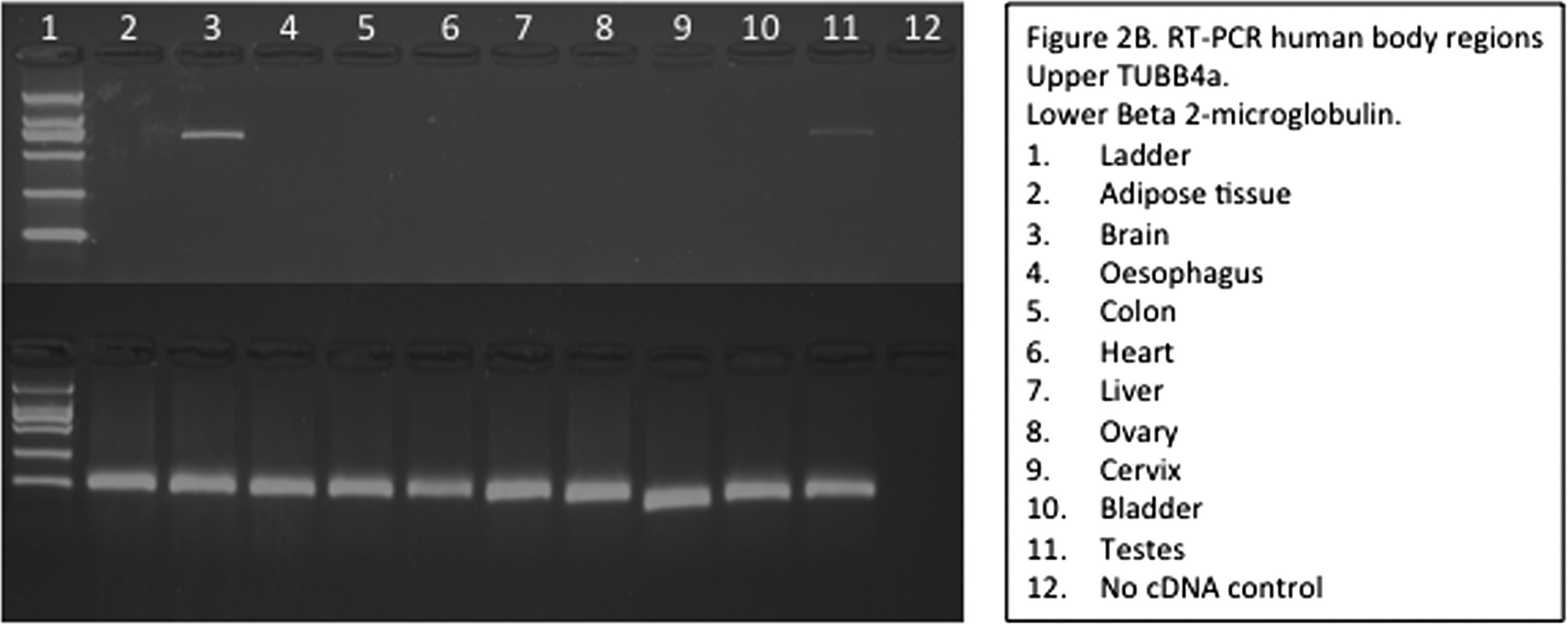
Expression of *TUBB4a* (top row) in various human tissues was determined by reverse transcriptase polymerase chain reaction using gene-specific primers against cDNA generated from tissue-specific RNA as compared to the housekeeping gene beta2-microglobulin (bottom row). Expression was high in the brain and very low in other tissues, except for moderate expression in the testes. 1 = ladder; 2 = adipose tissue; 3 = brain; 4 = esophagus; 5 = colon; 6 = heart; 7 = liver; 8 = ovary; 9 = cervix; 10 = bladder; 11 = testes; 12 = no cDNA control.

Expression of *TUBB4a* gene in 10 brain regions from 134 normal individuals was assessed using Affymetrix (Santa Clara, CA) Exon 1.0 ST Arrays, which identified high expression in the brain. These brains were negative for defects in the *TUBB4a* gene using Sanger sequencing. The highest expression was in the cerebellum, followed by putamen and white matter. There was a 2-fold difference between the cerebellum and the thalamus, with the lowest brain expression (Fig 4, Table 5). Expression of *TUBB4a* in other body tissues was very low except for moderate expression in the testes (Fig 5).

**TABLE 5:** Expression of *TUBB4a* in 10 Brain Regions from 134 Normal Individuals Assessed Using the Affymetrix Exon 1.0 ST Array

Region Sampled	Brain Regions, No.	Mean *TUBB4a* Expression
CRBL	130	10.84

FCTX	127	10.2

HIPP	122	10.2

MEDU	119	10.47

OCTX	129	10.19

PUTM	129	10.18

SNIG	101	9.94

TCTX	119	10.09

The values for *TUBB4a* expression are corrected for brain bank, batch effect, and gender effects. See Trabzuni et al for detailed methods.^23^

CRBL = cerebellum; FCTX = frontal cortex; HIPP = hippocampus; MEDU = medulla; OCTX = occipitalcortex; PUTM = putamen; SNIG = substantia nigra; TCTX = temporal cortex.

## Discussion

We have demonstrated through a combination of genetic linkage analysis, exome sequencing, and expression studies that the causative mutation in the DYT4 kindred is a heterozygous missense c.4C>G p.R2G *TUBB4a* mutation in exon 1. The location of this mutation within the gene is highly significant, as it is within the autoregulatory MREI domain of the *TUBB4a* sequence. All β-tubulins contain this MREI domain, at the first 4 amino acid positions, and this is highly conserved throughout all eukaryotic cells. We also show that *TUBB4a* is highly expressed in all brain regions (see Fig 4), particularly the cerebellum, which is thought to have a central role in the pathogenesis of dystonia.^31^,^32^ The combination of linkage analysis and exome sequencing has previously been successful in identifying other dystonia genes such as *CIZ1*.^17^

Tubulin is a globular protein and the main constituent of microtubules, a major cytoskeletal component. Tubulins are formed from heterodimers of α and β subunits and are expressed in all eukaryotic cells.^33^ Multiple isotypes are present with a high degree of homology, differing only at the C-terminal domain, and are differentially expressed according to tissue type.^34^ The MREI tetrapeptide sequence at the start of the N-terminal domain has been demonstrated to be necessary for the autoregulation of the β-tubulin mRNA transcript.^35^ Autoregulated instability of tubulin mRNA is a regulatory mechanism whereby tubulin mRNA is degraded by an as yet unknown mechanism involving an interaction with the MREI domain of the nascent tubulin peptide as it emerges from the ribosome.^36^ It has been hypothesized that such a regulatory mechanism has evolved to ensure a stoichiometric balance of α and β subunits.^36^

Site-directed mutagenesis has been used to demonstrate the effect of amino acid substitutions, including the DYT4 c.4C>G p.R2G mutation, on tubulin mRNA autoregulation compared with the wild-type arginine at residue 2.^37^ It was shown that peptides with the wild-type MREI sequence retain their autoregulatory ability and lead to destabilization of β-tubulin mRNA following elevation of intracellular tubulin subunit concentration. Alternate amino acids at this position, including p.R2G, abrogate this autoregulatory capability with no reduction in mRNA levels seen following an increase in tubulin subunit levels.^28^ In addition, the wild-type arginine at residue 2 prevents cleavage of the terminal methionine, whereas glycine promotes its removal,^38^ which may further impair the autoregulatory function of unpolymerized β-tubulin monomers.

The DYT4 family investigated here is the largest reported in the literature stretching back over many generations with this unusual phenotype. Other smaller kindreds have been reported with similar clinical features that in addition have similarities to some DYT6 families. We screened a number of autosomal dominant dystonia families and dystonia brains collected at our institute for *TUBB4A* mutations, and although no other mutations were identified, it will be important to analyze this gene in other similar pedigrees. A number of human diseases are caused by heterozygous mutations in several genes encoding α and β isotypes. Missense mutations in *TUBA1A* (class 1a α-tubulin), *TUBB2B* (class 2b β-tubulin), and *TUBB3* (class 3 β-tubulin) have all been reported and result in a range of severe neurological manifestations.^38^,^39^

There are few common molecular pathways that have emerged to link the dystonia-related genes and the clinical outcome. The cytoskeleton has a key role in coordinating the interactions between the transmembrane proteins of the inner and outer membrane that connect and position nuclei to the cytoplasmic cytoskeleton. In DYT1, mutant *TOR1A* interferes with the nucleocytoskeletal network, possibly by restricting movement of these particles/filaments, and hence this may affect development of neuronal pathways in the brain.^40^,^41^ Mutant *TUBB4a* could act through a similar pathway to *TOR1A*, with impaired tubulin autoregulation, which may result in a stoichiometric imbalance of α and β tubulin subunits and aberrant cytoskeletal binding.^42^ Further work on this interaction will be important to define the role of *TUBB4a,* the cytoskeleton, and the potential interactions with other dystonia disease genes.
