# Supplementary material for: Mutations in the Autoregulatory Domain of β-Tubulin 4a Cause Hereditary Dystonia
Source: Ann Neurol. 2013 Feb 19;73(4):546–53. doi: 10.1002/ana.23832 (PMC3698699; doi:10.1002/ana.23832)
Supplement: Supplementary file 1 [file ana0073-0546-SD1.doc]

**Supplementary methods**

Samples – Blood samples were collected and DNA extracted with informed consent, from England and Australia, (ethics approval 06/N076) from 38 family members (marked on pedigree with m/wt on Figure 1). Clinical details of selected affected family members were obtained through direct patient interview and clinical examination and review of historical records. These data are given in Table 1[1-3](http://mc.manuscriptcentral.com/LongRequest/ana?TAG_ACTION=DOWNLOAD_FILE_BY_NAME&DOCUMENT_ID=12809382&FILE_TO_DOWNLOAD=12809382_File000006_245949245.html-withlinks.htm&FILE_KEY=-1302593310&FILE_NAME_KEY=-992827567&DOWNLOAD=TRUE&FILE_TYPE=DOCUMENT&DOCUMENT_HASHCODE=811728253&SANITY_CHECK_DOCUMENT_ID=12809382&CURRENT_ROLE_ID=1964#_ENREF_1). Exome sequencing was performed on two cases (VI-2 and VII-6). An additional 124 UK dystonia families and 75 dystonia brains cases were selected for further analysis; these cases were negative for DYT1 and DYT6. This mutation was absent from 1045 UK control individuals and absent in 7203 exomes from the UCL and NHLBI exome sequencing projects.

Genetic linkage analysis – This was carried out on 19 family members (marked on pedigree with ❖ in Figure 1), comprising 10 unaffected and 9 affected cases. These were genotyped using Illumina CytoSNP12 arrays with 301,232 genome-wide markers and the raw data was processed in GenomeStudio (Illumina, San Diego, US). Genotypes were examined with the use of a multipoint parametric linkage analysis and haplotype reconstruction performed with Simwalk2 ^8^. 24,000 informative SNPs, equally spaced 0.1cM apart were used in the analysis. Data were formatted with Mega2 ^9^ via ALOHMORA ^10^. Mendelian inconsistencies were checked with PedCheck ^11^. An autosomal dominant model was specified with an estimated allele frequency of 0.00001 and 90% penetrance. The linkage region was subsequently used to filter the variants obtained from exome sequencing.

Exome sequencing - Libraries were prepared using the TruSeq DNA sample prep kit (Illumina, San Diego, US) and 1ug of patient DNA. Fragmentation was performed using the Covaris shearing system using the 300bp with size selection by excision from the area between 300-400bp on an agarose gel. Exome capture was performed using the TruSeq Exome enrichment kit using 750ng of prepared library as input. The samples were then sequenced using the version 3, HiSeq 2000 flow-cell and sequencing kit on the HiSeq 2000 platform. Data was demultiplexed using the CASAVA tool and reads aligned to the NCBI human reference genome hg19 using Novoalign (Novocraft, Sengalor, Malaysia). Duplicate reads were removed using Picard (sourceforge.net/projects/picard) with on-target statistics generated with Picard tools. Variant calling including indels was performed using SAMtools (samtools.sourceforge.net) with the resulting variant files annotated with ANNOVAR ([www.openbioinformatics.org/annovar](http://www.openbioinformatics.org/annovar)). Annotated variant files were generated which included a comparison to several reference databases including dbSNP (www.ncbi.nlm.nih.gov/projects/SNP), 1000genomes (www.1000genomes.org), NHLBI Exome Variant Server (evs.gs.washington.edu) and Complete Genomics cg69 database (www.completegenomics.com/public-data/69-Genomes). In-silico predictions of pathogenicity were performed using SIFT[4](http://mc.manuscriptcentral.com/LongRequest/ana?TAG_ACTION=DOWNLOAD_FILE_BY_NAME&DOCUMENT_ID=12809382&FILE_TO_DOWNLOAD=12809382_File000006_245949245.html-withlinks.htm&FILE_KEY=-1302593310&FILE_NAME_KEY=-992827567&DOWNLOAD=TRUE&FILE_TYPE=DOCUMENT&DOCUMENT_HASHCODE=811728253&SANITY_CHECK_DOCUMENT_ID=12809382&CURRENT_ROLE_ID=1964#_ENREF_4), PolyPhen2[5](http://mc.manuscriptcentral.com/LongRequest/ana?TAG_ACTION=DOWNLOAD_FILE_BY_NAME&DOCUMENT_ID=12809382&FILE_TO_DOWNLOAD=12809382_File000006_245949245.html-withlinks.htm&FILE_KEY=-1302593310&FILE_NAME_KEY=-992827567&DOWNLOAD=TRUE&FILE_TYPE=DOCUMENT&DOCUMENT_HASHCODE=811728253&SANITY_CHECK_DOCUMENT_ID=12809382&CURRENT_ROLE_ID=1964#_ENREF_5) and MutationTaster[6](http://mc.manuscriptcentral.com/LongRequest/ana?TAG_ACTION=DOWNLOAD_FILE_BY_NAME&DOCUMENT_ID=12809382&FILE_TO_DOWNLOAD=12809382_File000006_245949245.html-withlinks.htm&FILE_KEY=-1302593310&FILE_NAME_KEY=-992827567&DOWNLOAD=TRUE&FILE_TYPE=DOCUMENT&DOCUMENT_HASHCODE=811728253&SANITY_CHECK_DOCUMENT_ID=12809382&CURRENT_ROLE_ID=1964#_ENREF_6). Protein sequence alignment was performed using ClustalOmega (www.ebi.ac.uk/Tools/msa/clustalo).

*TUBB4a* mutation screening - To validate the results of exome sequencing, to sequence other dystonia families and control individuals, primers were designed using Primer3 to amplify the entire *TUBB4a* gene (primers available on request). The following reaction conditions were used for PCR amplification: 12µl FastStart Mastermix (Roche), 1µl (10pM) forward and reverse primers, 1µl DMSO, 4µl PCR grade water, 1µl DNA (30ng/µl). PCR products were visualized on a 2% agarose gel and cleaned with Millipore filter plates. Clean PCR product was sequenced using BigDye Terminator 3.1 chemistry (Life Technologies, Carlsbad, CA, USA) and cleaned with Millipore filter plates prior to capillary electrophoresis on an ABI 3130XL Genetic Analyzer (ABI Biosystems, Foster City, CA, USA). Sequencing data was visualized using Sequencher software (Gene Codes Corporation, Ann Arbor, MI, USA). The genotype of the family members screened is indicated on the pedigree as either *w* (wildtype) or *m* (mutant - R2G heterozygote).

Expression methods – Regional distribution of *TUBB4a* mRNA expression in the normal human brain was determined using microarray analysis of human post-mortem brain tissue from the UK Human Brain Expression Consortium[7](http://mc.manuscriptcentral.com/LongRequest/ana?TAG_ACTION=DOWNLOAD_FILE_BY_NAME&DOCUMENT_ID=12809382&FILE_TO_DOWNLOAD=12809382_File000006_245949245.html-withlinks.htm&FILE_KEY=-1302593310&FILE_NAME_KEY=-992827567&DOWNLOAD=TRUE&FILE_TYPE=DOCUMENT&DOCUMENT_HASHCODE=811728253&SANITY_CHECK_DOCUMENT_ID=12809382&CURRENT_ROLE_ID=1964#_ENREF_7). Brain tissues originating from 134 control Caucasian individuals were collected by the Medical Research Council (MRC) Sudden Death Brain and Tissue Bank (Edinburgh, UK). The following brain regions were included in the analysis: cerebellum (CRBL), frontal cortex (FCTX), hippocampus (HIPP), medulla (MEDU), occipital cortex (OCTX), putamen (PUTM), substantia nigra (SNIG), temporal cortex (TCTX), thalamus (THAL) and white matter (WHMT). Total RNA was isolated from these tissues using mRNeasy 96-well kit (Qiagen, UK) before processing with the Ambion WT Expression Kit and Affymetrix GeneChip Whole Transcript Sense Target Labeling Assay, and hybridization to the Affymetrix Exon 1.0 ST Array. The probeset defining TUBB4a mRNA was determined using the Affymetrix Netaffx annotation file (HuEx-1_0-st-v2 Probe set Annotations, Release 31). The combined signal of the TUBB4a probe sets was used to determine mRNA expression.

Expression of TUBB4a in various human tissues was determined using gene-specific primers against cDNA generated from tissue-specific RNA in the FirstChoice Human Total RNA Survey Panel (Life Technologies, Carlsbad, USA). The cDNA was synthesised from the mRNA panel with SuperScript II reverse transcriptase according to the manufacturer’s protocol, 1000ng of mRNA was used as template with random oligonucleotide primers. cDNA was made from the following regions, trachea, thyroid, prostate, skeletal muscle, spleen, small intestine, thymus, lung, placenta, kidney, adipose tissue, brain, oesophagus, colon, heart, liver, ovary, cervix, bladder and testes. One microliter of the resulting cDNA product was then used as a template for the RT-PCR reaction at 30 cycles with primers (forward CTCGCCTGCACTTCTTCAT and reverse CAGCCTCTTTGTTCCCAGTC) specific to *TUBB4a* cDNA and a comparative reaction with a housekeeping gene. This was visualised on a 2% agarose gel.

**Supplementary method references**

1. Parker N. Hereditary whispering dysphonia. Journal of neurology, neurosurgery, and psychiatry. 1985 Mar;48(3):218-24.

2. Waddy HM, Fletcher NA, Harding AE, Marsden CD. A genetic study of idiopathic focal dystonias. Annals of neurology. 1991 Mar;29(3):320-4.

3. Wilcox RA, Winkler S, Lohmann K, Klein C. Whispering dysphonia in an Australian family (DYT4): a clinical and genetic reappraisal. Movement disorders : official journal of the Movement Disorder Society. 2011 Nov;26(13):2404-8.

4. Kumar P, Henikoff S, Ng PC. Predicting the effects of coding non-synonymous variants on protein function using the SIFT algorithm. Nature protocols. 2009;4(7):1073-81.

5. Adzhubei IA, Schmidt S, Peshkin L, et al. A method and server for predicting damaging missense mutations. Nature methods. 2010 Apr;7(4):248-9.

6. Schwarz JM, Rodelsperger C, Schuelke M, Seelow D. MutationTaster evaluates disease-causing potential of sequence alterations. Nature methods. 2010 Aug;7(8):575-6.

7. Trabzuni D, Ryten M, Walker R, et al. Quality control parameters on a large dataset of regionally dissected human control brains for whole genome expression studies. Journal of neurochemistry. 2011 Oct;119(2):275-82.

8. Sobel E, Sengul H, Weeks DE (2001) **Multipoint estimation of identity-by-descent probabilities at arbitrary positions among marker loci on general pedigrees**. Human Heredity 52:121-131.

9. Baron RV, Kollar CP, Mukhopadhyay N, Almasy L, Schroeder M, Mulvihill WP, Weeks DE (2012) Mega2 (Version 4.5.6).http://watson.hgen.pitt.edu

10. Ruschendorf F, Nurnberg P. ALOHOMORA: a tool for linkage analysis using 10K SNP array data Bioinformatics. 2005 May 1; 21(9): 2123-5.

11. O’Connell JR, Weeks DE, “PedCheck: A program for identifying genotype incompatibilities in linkage analysis, Am J Hum Genet 63:259-266.
